# Development and validation of an instrument to assess the prescribing readiness of medical students in Malaysia

**DOI:** 10.1186/s12909-015-0433-z

**Published:** 2015-09-21

**Authors:** Pauline Siew Mei Lai, Si Mui Sim, Siew Siang Chua, Choo Hock Tan, Chirk Jenn Ng, Francis Ifejika Achike, Cheong Lieng Teng

**Affiliations:** 1 Department of Primary Care Medicine, University of Malaya Primary Care Research Group (UMPCRG), Faculty of Medicine, University of Malaya, 50603 Kuala Lumpur, Malaysia; 2 Department of Pharmacology, Faculty of Medicine, University of Malaya, 50603 Kuala Lumpur, Malaysia; 3 Department of Pharmacy, Faculty of Medicine, University of Malaya, 50603 Kuala Lumpur, Malaysia; 4 Division of Clinical Science, School of Medicine and Allied Health Sciences, International Medical University, 57000 Kuala Lumpur, Malaysia; 5 Central Michigan University College of Medicine, Mount Pleasant, MI 48859 USA

## Abstract

**Background:**

Prescribing incompetence is an important factor that contributes to prescribing error, and this is often due to inadequate training during medical schools. We therefore aimed to develop and validate an instrument to assess the prescribing readiness of medical students (PROMS) in Malaysia.

**Methods:**

The PROMS comprised of 26 items with four domains: undergraduate learning opportunities; hands-on clinical skills practice; information gathering behaviour; and factors affecting the learning of prescribing skills. The first three domains were adapted from an existing questionnaire, while items from the last domain were formulated based on findings from a nominal group discussion. Face and content validity was determined by an expert panel, pilot tested in a class of final year (Year 5) medical students, and assessed using the Flesch reading ease. To assess the reliability of the PROMS, the internal consistency and test-retest (at baseline and 2 weeks later) were assessed using the Wilcoxon Signed Ranks test and Spearman’s rho. The discriminative validity of the PROMS was assessed using the Mann–Whitney U-test (to assess if the PROMS could discriminate between final year medical students from a public and a private university).

**Results:**

A total of 119 medical students were recruited. Flesch reading ease was 46.9, indicating that the instrument was suitable for use in participants undergoing tertiary education. The overall Cronbach alpha value of the PROMS was 0.695, which was satisfactory. Test-retest showed no difference for 25/26 items, indicating that our instrument was reliable. Responses from the public and private university final year medical students were significantly different in 10/26 items, indicating that the PROMS was able to discriminate between these two groups. Medical students from the private university reported fewer learning opportunities and hands-on practice compared to those from the public university. On the other hand, medical students from the private university reported more frequent use of both web based and non-web-based resources compared to their public university counterparts.

**Conclusions:**

The PROMS instrument was found to be a reliable and valid tool for assessing medical students’ readiness to prescribe in Malaysia. It may also inform on the adequacy of medical programmes in training prescribing skills.

**Electronic supplementary material:**

The online version of this article (doi:10.1186/s12909-015-0433-z) contains supplementary material, which is available to authorized users.

## Background

Prescribing is a fundamental skill in medical practice. Nevertheless, concerns have continuously been expressed in many parts of the world, including Malaysia, about the preparedness of medical students for entry into the complex and challenging environment of prescribing [1–4]. Previous studies in Malaysia showed that there was a low rate of compliance by medical practitioners to the legal and procedural requirements in prescription writing [5, 6]. Another study found that there was insufficient opportunity for medical students to practice prescribing [7]. The medical curriculum has therefore become the focus of this phenomenon for various reasons, including the reduced visibility of clinical pharmacology and therapeutics in the curriculum [8, 9]. A lack of standardisation in the teaching of prescribing skills across the undergraduate medical curriculum may be the other reason [7].

Throughout the world, new medical graduates are required to undergo internship, a period of medical apprenticeship under the supervision of clinical consultants. However, interns have been found to be involved in a number of prescribing errors [10–13]. This may be due to the inadequate or irrelevant teaching of clinical pharmacology [2, 14]; indicating that undergraduate training may be inadequate for meeting subsequent clinical work demands. Clear guidelines on undergraduate medical curriculum has been given by the General Medical Council (GMC) of the United Kingdom in Tomorrow’s Doctors 2009 on the expected minimal outcomes with regards to drug therapy (such as an understanding of adverse effects and harmful drug interactions) [15]. This has impacted upon the revision of the undergraduate medical curriculum around the globe, including the University of Edinburgh, where a vertical theme of clinical pharmacology and therapeutics was emphasized [16]. In many medical schools in Asia (including our public university), pharmacology is typically taught in the second year (the “para-clinical” year), mainly via lectures and practical sessions, while a structured programme on prescribing is conspicuously lacking [17]. It appears that students only learn about ‘prescribing’ in a rather ‘*ad hoc*’ fashion during their clinical postings or clerkships. In addition, prescribing is often perceived as only ‘the writing of drug on a prescription sheet’ instead of an appreciation of the whole process of good prescribing as encompassing essential aspects like information gathering, decision making, patient counselling, and monitoring, in accordance with the World Health Organization 6-step model [18]. Recently, Nazar et al. (2015) developed a framework to teach safe and effective prescribing (based on the 4Ps) within the UK undergraduate medical curriculum that can be subsumed onto the GMC learning outcomes [19].

Studies from different countries have assessed medical graduates’ readiness for workplace/clinical practice, particularly their prescribing competence and the factors or problems associated with their preparedness. The assessment methods and scopes varied from one study to another. Most studies used quantitative methods such as self-reporting structured questionnaires [11, 16, 20–25], or objective structured clinical examination (OSCE) [26], or real patient consultations on clinical practice [27]. Other studies used qualitative methods [28–30]. The scopes of assessment of these readiness-to-practice studies also varied from knowledge of pharmacology to more practical applications like the personal drug (P-drug) selection process, information gathering, stress-coping strategies, instructing/educating patients, and communication skills [18]. It is our observation that not many studies on medical students preparedness for workplace clinical practice and/or prescribing have been conducted in Asia, which has witnessed a rapid expansion in the number of medical schools in the last decade.

Hans and Maxwell [16] developed a questionnaire to assess the readiness to prescribe for their new graduates, i.e., foundation year 1 doctors, whereas we wanted an instrument to assess the readiness of medical students to prescribe upon graduation. Furthermore, the questionnaire developed by them did not report that it had been validated. Therefore, the aim of our study was to develop and validate an instrument to assess the prescribing readiness of medical students (PROMS). We hope that this instrument would serve as a good reference for other medical schools which intend to review their prescription writing curriculum.

## Methods

### Study design

The PROMS was developed in February-March 2008, and validated in April-May 2013, at the University of Malaya (a public university) and at the International Medical University (a private university), Malaysia.

The study was divided into two parts: the development of an instrument used for assessing the prescribing readiness of medical students and its validation.

### Part 1: Development of the PROMS instrument

#### The instrument development process

We extracted 16 items from section 1 of the questionnaire developed by Han and Maxwell [16] in their study on foundation year 1 doctors (i.e., first-year interns or house-officers). These 16 items were grouped into 3 domains: (1) undergraduate (i.e., pre-licensure) learning opportunities, (2) hands-on clinical skills practice, and (3) information gathering behaviour. We used only these selected questions from the published questionnaire (permission obtained by personal communication) as they were the only questions that were related to an undergraduate’s training experience.

We developed a fourth domain: factors affecting prescribing skills acquisition, by using the nominal group technique (NGT) among nine final year (Year 5) medical students (5 females, 4 males) in a public university in Malaysia. One major advantage of using the NGT is that it ensures relatively equal participation by all members in the decision making process. The NGT consists of five stages: (1) the facilitator gave a brief introduction and explanation to the group on the purpose and procedure of the meeting. (2) Each group member was asked to write down all ideas that came to mind in response to: “What are the factors that affect your learning in acquiring prescribing skills?” During this period, members were asked not to consult or discuss their ideas with others. (3) Each group member took turns to share the ideas they had generated, one at a time, and these were recorded on a flip chart. The round robin process continued until all ideas had been presented. No debate or discussion of ideas occurred at this stage. (4) Group members were invited to seek clarification on any of the ideas that fellow members had produced. At this stage the wording of any unclear statements was clarified, similar ideas were combined to form hybrid ideas, and new ideas were allowed to be put forward, but no ideas were eliminated. (5) This stage involved prioritising the recorded ideas in relation to the original question. Each group member scored the recorded ideas/factors independently using a 10 point Likert-like scale (1 = least important; 10 = most important) and the scores were weighted for each member. The factor (idea) with the highest weighted score by the group was considered the most important by this group consensus, and a list of the ideas in the order of their ranked importance to the group was produced. Out of the 17 factors generated, the factors with the ten highest scores were selected to be included in the PROMS instrument. Although it would be ideal to have several NGTs to capture all the possible factors, we believe that one NGT has managed to identify the most crucial factors.

### Face and content validity

The final version of the PROMS was reviewed by a panel of experts, which consisted of two pharmacists, two clinicians and two pharmacologists. We then administered this questionnaire to the entire class, with an option for students to remark if there were additional factors we had missed. The findings of this pilot study revealed no additional new factors. Hence, no further modifications were made to the PROMS.

### Final version of the instrument

The finalized instrument consisted of two sections with four domains (Additional file 1). Section A contained three domains that encompassed the 16 items on “your undergraduate training experience on drugs and prescribing”, while section B contained one domain that included the 10 items on “factors affecting your learning of prescribing skills.” For section A, participants selected their responses according to a set of five descriptors (e.g., “far too little”, “too little”, “just right”, “too much”, “far too much” or “never”, “1–5”, “6–10”, “11–15”, “>15” or “never”, “yearly”, “monthly”, “weekly”, “daily”) given for each domain. For section B, there was a 5-point Likert-like scale, where 1 represented “strongly disagree” and 5 represented “strongly agree” Participants took 10–15 min to answer the questionnaire.

### Part II: Validation of the PROMS instrument

#### Subjects

Included were final year (Year 5) medical students from a public and a private university in Kuala Lumpur, Malaysia.

### Sample size

The number of students used to validate this tool was calculated based on an item to participant ratio of 5:1. Since there were 16 items in Section A of the PROMS instrument that could be validated, the total number of participants required was 80.

### Data collection

Final year students were recruited using convenience sampling. The purpose of the study was explained to them after one of their large class sessions. For those who agreed to participate, informed consent was obtained. The PROMS was administered to the entire class of final year medical students, and was therefore representative of the entire cohort. Their participation was voluntary, with no reward. Participants were asked to respond to each statement based on their own experience or perception.

### Reliability

The PROMS was administered at baseline and two weeks later to assess for test-retest reliability.

### Discriminative validity

To determine if the PROMS was able to discriminate between responses from different medical students, it was administered to a class of final year (year 5) medical students in a private medical school located in the vicinity of the public medical school under study. These two medical schools’ main campuses are both located in the same city with comparable teaching facilities and faculty strength, although their clinical year students undertake rotational clinical clerkship in different government hospitals at different parts of the country. English is the main medium of instruction in both medical schools. However, there may be differences with respect to intake criteria, entrance requirements, socio-economic status of students and curriculum delivery. Indeed, there were distinct differences in how basic pharmacology, clinical pharmacology and pharmacotherapy were taught in these two universities. The private university used a full problem-based learning (PBL) approach, while the public university used a hybrid PBL curriculum - where the teaching of pharmacology was still predominantly by didactic lectures in Year 2. The private university introduced clinical teaching much earlier on (from Year 1) in their medical programme whereas clinical exposure started only in Year 2 in the public university. Students in this private university are mostly self-funded, whilst students in the public university are generally government funded. We hypothesized that the responses provided by the students from these public and private universities would be different.

### Ethics approval

Ethics approval for this study was granted by the Medical Ethics Committee of the University of Malaya Medical Centre (approval number: 667.4). Informed consent was obtained from all participants prior to the study.

### Statistical analyses

All data was entered into IBM® SPSS® version 22 (IBM Corporation, Armonk, NY, USA). Descriptive statistics were presented as percentage and frequencies, while means and standard deviations were calculated for continuous variables. Associations between categorical variables were analysed using chi square tests while t-tests were used for continuous variables.

### Face and content validity

The Flesch reading index was calculated for the PROMS using Microsoft Office® Word® 2007 (Microsoft Corporation, Redmond, WA, USA). This was to determine the reading comprehension level necessary to answer the questions. Calculation is based on the average number of syllables per word and the average number of words per sentence. Scores range from zero to 100, with lower numbers indicating greater difficulty. An average document should have a score of 60–70 [31]. The Kolmogorov-Smirnov test was used to determine if the data were normally distributed.

### Reliability

#### Internal consistency

The overall internal consistency (Cronbach’s alpha) was calculated for the first three domains: “undergraduate learning opportunities”, “hands-on clinical skills practice” and “information gathering behaviour”, as these three domains assessed prescribing readiness. The domain: “factors affecting prescribing skills acquisition” was excluded as these items were merely descriptive in nature. A Cronbach’s alpha value of 0.5-0.69 is acceptable, while values of 0.70-0.90 indicate strong internal consistency [32].

The corrected item-total correlations show the extent with which each item in the instrument is correlated to the total score. Corrected item-total correlations should be >0.3 for it to be considered as acceptable [33].

### Test-retest reliability

This was assessed using the Wilcoxon Signed Ranks test and Spearman’s rho, as normality could not be assumed. Correlations were interpreted as follows: little or no correlation (0–0.25), fair correlation (>0.25-0.5), moderate to good correlation (>0.5-0.75) and very good to excellent correlation (>0.75) [34].

### Discriminative validity

The Mann–Whitney U-test was used to determine if there was any difference in responses obtained from medical students of a public and a private university. A p-value of < 0.05 was considered as statistically significant.

## Results

A total of 119 students were approached, and 119 students answered the questionnaire (response rate = 100 %). The majority of our students were female [77 (64.7 %)], with a mean age ± SD = 24.0 ± 0.6 years [range: 22–26 years], and predominantly of Chinese [58 (48.7 %)] and Malay [55 (46.2 %)] ethnicity; the remaining 5.1 % consisted of ethnic Indians and others.

### Face and content validity

Flesch reading ease was 46.9.

### Reliability

#### Internal consistency

The overall Cronbach’s alpha value for the PROMS was 0.695. The Cronbach’s alpha values for the first three domains ranged from 0.542–0.736. Corrected item-total correlations showed that all items were >0.30, except for items no.1, 2, 9, 10, 15 and 16. However, for items 9, 10, 15 and 16, the removal of these items did not improve the Cronbach’s alpha. The removal of items 1 and 2 however, did increase the Cronbach’s alpha, but not by a significant amount. Hence, all 16 items were retained [Table 1].

**Table 1:** The psychometric properties of the prescribing readiness of medical students (PROMS)

		Test (*n* = 119)		Retest (*n* = 119)		Wilcoxon Sign Rank test		Spearman’s rho correlation‡	Corrected item-total correlation	Cronbach’s α if item is deleted	Cronbach alpha
Domain	Item	Mean (SD)	Median	Mean (SD)	Median	Mean rank	*p*-value ‡				
Undergraduate learning opportunities	1. Lectures on the basic pharmacology of drugs	2.93 (0.41)	3.00	2.92 (0.42)	3.00	14.08	0.847	0.233	0.234	0.751	0.736
						12.00					
	2. Lectures on the use of drugs in clinical practice	2.53 (0.59)	3.00	2.53 (0.62)	3.00	21.66	0.982	0.416	0.235	0.759	
						19.45					
	3. Small group tutorials about drugs and prescribing	2.05 (0.64)	2.00	2.12 (0.67)	2.00	20.08	0.338	0.409	0.591	0.664	
						22.67					
	4. Problem based learning about drugs and prescribing	2.13 (0.67)	2.00	2.13 (0.67)	2.00	25.92	0.912	0.312	0.602	0.658	
						25.08					
	5. Workshops on prescribing issues	1.87 (0.66)	2.00	1.98 (0.69)	2.00	24.08	0.094	0.366	0.664	0.639	
						25.58					
	6. Electronic learning opportunities	1.98 (0.77)	2.00	2.10 (0.76)	2.00	28.75	0.169	0.268	0.513	0.690	
						30.03					
Hands-on clinical skills practice	7. Write up a hospital drug cardex	1.56 (0.68)	1.00	1.63 (0.72)	2.00	19.50	0.252	0.431	0.336	0.474	0.542
						22.17					
	8. A teaching session on calculating drug doses	1.58 (0.66)	2.00	1.67 (0.64)	2.00	19.96	0.087	0.523	0.373	0.458	
						20.02					
	9. Set up a drug infusion pump	1.28 (0.50)	1.00	1.55 (0.79)	1.00	19.50	<0.001*	0.549	0.206	0.537	
						18.92					
	10. Prepare and give a parenteral drug injection	2.51 (1.15)	2.00	2.49 (1.10)	2.00	29.88	0.857	0.528	0.282	0.540	
						27.30					
	11. Set up and give a bag of intravenous fluid	1.93 (0.90)	2.00	1.95 (0.90)	2.00	17.56	0.769	0.660	0.410	0.414	
						18.42					
Information gathering behaviour	12. Web based resources	2.97 (1.24)	3.00	2.81 (1.05)	3.00	32.95	0.158	0.495	0.353	0.563	0.610
						34.35					
	13. Online medical school resources	3.08 (1.14)	3.00	3.23 (1.12)	3.00	31.74	0.141	0.506	0.539	0.458	
						33.89					
	14. Your own textbook	3.42 (1.27)	4.00	3.49 (1.23)	4.00	27.30	0.608	0.589	0.370	0.554	
						30.64					
	15. Your own BNF or equivalent	1.96 (1.11)	1.00	1.93 (1.14)	1.00	21.77	0.773	0.536	0.289	0.593	
						25.75					
	16. Library resources	1.43 (0.81)	1.00	1.42 (0 .77)	1.00	17.30	0.930	0.438	0.296	0.590	
						15.79					
Factors affecting prescribing skills acquisition	17. No lecture or formal teaching on prescribing drugs	3.17 (1.12)	3.00	3.21 (0.91)	3.00	30.14	0.923	0.341			
						34.05					
	18. Amount of knowledge on drugs needed to be learnt is too much during each clinical posting	3.08 (1.02)	3.00	2.87 (0 .99)	3.00	36.54	0.065	0.333			
						31.59					
	19. Will not be questioned on prescribing drugs in the final examination	2.09 (0.86)	2.00	2.03 (0 .80)	2.00	23.08	0.473	0.571			
						22.90					
	20. Many clinical teachers do not explain about their rationale for their choice of drugs prescribed	2.75 (0.95)	3.00	2.80 (0.84)	3.00	29.28	0.454	0.507			
						32.56					
	21. Not enough of reinforcement of pharmacology knowledge in the clinical years	3.13 (0.95)	3.00	3.15 (0.84)	3.00	28.50	0.804	0.506			
						31.55					
	22. Not necessary for me to know how to prescribe drugs before graduation	1.99 (0.93)	2.00	1.97 (0.91)	2.00	24.68	0.750	0.544			
						24.30					
	23. Lack of consensus among clinical teachers on drug prescribing	2.61 (0.79)	3.00	2.73 (0.77)	3.00	25.64	0.140	0.422			
						27.08					
	24. Preclinical learning on pharmacology didn’t have enough clinical relevance	3.34 (1.11)	3.00	3.33 (1.02)	3.00	34.23	0.880	0.593			
						36.92					
	25. No actual practice, experience or emphasis on prescribing in clinical years	3.48 (0.95)	3.00	3.42 (0.94)	3.00	32.71	0.431	0.576			
						31.17					
	26. Limited exposure to information that can help in making rational drug choice	3.03 (1.00)	3.00	2.89 (0.89)	3.00	36.26	0.096	0.540			
						27.00					

*Statistically significant at *p* < 0.05

‡All Spearman’s rho correlation values were statistically significant at *p* < 0.05

### Test-retest

Test-retest reliability was assessed in 119 participants after a 2-week interval and 25/26 items showed no difference at test-retest. Fourteen out of 26 items (53.8 %) had moderate to good correlation, 11/26 items (42.3 %) had fair correlation, whilst only 1/26 item (3.8 %) had little or no correlation [Table 1].

### Discriminative validity

A total of 176 final year (year 5) medical students were recruited: 123 (69.9 %) and 53 (30.1 %) from a public and a private university, respectively. No demographic differences were found between these two groups of students [Table 2].

**Table 2:** Demographic characteristics of final- year medical students from a public and a private university

	Public university (*n* = 123)	Private university (n = 53)	*p*-value ‡
Gender [n (%)]^a^			
Female	83 (67.5)	31 (58.5)	0.252
Male	40 (32.5)	22 (41.5)	
Mean age ± SD (years) [range]	24.2 ± 0.8 [23–29]	24.2 ± 1.1 [22–28]	0.943
Ethnicity [n (%)]^a^			
Malay	67 (54.5)	28 (52.8)	0.107
Chinese	52 (42.3)	19 (35.8)	
Indian	2 (1.6)	5 (9.4)	
Others	2 (1.6)	1 (1.9)	

*SD* Standard deviation

‡χ^2^ test was used for all categorical variables whilst the independent *t*-test was used for all continuous variables

^a^Data unavailable for 1 participant (gender), 1 participant (ethnicity)

Significant differences were found for 10/26 (38.5 %) items between responses of final year medical students from a public and those from a private university [Table 3]. Medical students from the private university reported that they had too little of lectures on the basic pharmacology of drugs, drugs used in clinical practice, and small group tutorials about drugs compared to medical students from the public university. Medical students from the private university also had significantly less opportunity to prepare and give a parenteral drug injection compared to medical students from the public university. On the other hand, medical students from the public university reported less frequent use of all the five listed resources, especially the library resources, compared to their counterparts from the private university.

**Table 3:** Prescribing readiness of final- year medical students in a public and a private university

		Public university (*n* = 123) [%]			Private university (*n* = 53) [%]			
Domain	Item	Too little	About right	Too much	Too little	About right	Too much	*p*-value
Undergraduate learning opportunities	1. Lectures on the basic pharmacology of drugs	10.6	83.7	5.7	42.6	51.9	5.6	<0.001*
	2. Lectures on the use of drugs in clinical practice	50.4	49.6	0	72.2	25.9	1.9	0.006*
	3. Small group tutorials about drugs and prescribing	72.4	26.8	0.8	90.7	9.3	0	0.024*
	4. Problem based learning about drugs	73.2	24.4	2.4	75.9	24.1	0	0.507
	5. Workshops on prescribing issues	74.6	24.6	0.8	85.2	14.8	0	0.267
	6. Electronic learning opportunities	68.6	27.3	4.1	65.4	34.6	0	0.238
		0–5 times	6–10 times	>10 times	0–5 times	6–10 times	>10 times	
Hands-on clinical skills practice	7. Write up a hospital drug cardex	90.2	8.1	1.6	84.9	9.4	5.7	0.314
	8. A teaching session on calculating drug doses	92.7	6.5	0.8	85.2	11.1	3.7	0.213
	9. Set up a drug infusion pump	92.7	6.5	0.8	92.6	7.4	0	0.785
	10. Prepare and give a parenteral drug injection	20.3	9.8	69.9	68.5	20.4	11.1	<0.001*
	11. Set up and give a bag of intravenous fluid	60.2	22.8	17.1	54.7	18.9	26.4	0.355
		Never or yearly	Monthly	Weekly or daily	Never or yearly	Monthly	Weekly or daily	
Information gathering behaviour	12. Web based resources	56.6	27.9	15.6	33.3	22.2	44.4	<0.001*
	13. Online medical school resources	48.8	35.8	15.4	33.3	31.5	35.2	0.011*
	14. Your own textbook	5.7	48.0	46.3	9.3	24.1	66.7	0.012*
	15. Your own BNF or equivalent	41.3	28.4	30.3	17.0	30.2	52.8	0.004*
	16. Library resources	82.1	13.7	4.3	31.4	33.3	35.3	<0.001*
	Factors	Strongly disagree/disagree	Somewhat agree	Agree/ strongly agree	Strongly disagree/ disagree	Somewhat agree	Agree/ strongly agree	
Factors affecting prescribing skills	17. No lecture or formal teaching on prescribing drugs	39.0	41.5	19.5	20.4	48.1	31.5	0.036*
	18. Amount of knowledge on drugs needed to be learnt is too much during each clinical posting	39.3	32.0	28.7	33.3	31.5	35.2	0.645
	19. Will not be questioned on prescribing drugs in the final examination	60.7	25.4	13.9	70.4	22.2	7.4	0.359
	20. Many clinical teachers do not explain about their rationale for their choice of drugs prescribed	30.3	40.2	29.5	40.7	40.7	18.5	0.230
	21. Not enough of reinforcement of pharmacology knowledge in the clinical years	12.3	50.0	37.7	20.8	41.5	37.7	0.310
	22. Not necessary for me to know how to prescribe drugs before graduation	62.6	26.0	11.4	72.2	22.2	5.6	0.355
	23. Lack of consensus among clinical teachers on drug prescribing	25.2	62.6	12.2	26.4	54.7	18.9	0.457
	24. Preclinical learning on pharmacology didn’t have enough clinical relevance	26.0	35.0	39.0	27.8	37.0	35.2	0.889
	25. No actual practice, experience or emphasis on prescribing in clinical years	10.6	43.1	46.3	22.2	42.6	35.2	0.095
	26. Limited exposure to information that can help in making rational drug choice	40.7	43.9	15.4	31.5	46.3	22.2	0.394

*Statistically significant at *p* < 0.05

Most students agreed with the factors listed in the fourth domain of the PROMS. The top three factors were: (1) no actual practice, experience or emphasis on prescribing in clinical years, (2) preclinical learning on pharmacology didn’t have enough clinical relevance, and (3) no lecture or formal teaching on prescribing drugs.

## Discussion

The study showed that the first three domains (Section A) in the PROMS instrument formed a reliable and valid tool for assessing medical students’ readiness to prescribe in Malaysia. In addition, the fourth domain (Section B) of the PROMS identified the major factors affecting prescribing skills acquisition by medical students.

The PROMS instrument had a Flesch reading ease of 46.9, indicating that this instrument is more difficult to read than a standard document which should have a Flesch reading ease of 60–70 (that is easily understood by 13–15 year old students). However, the PROMS was still suitable for use among medical undergraduates who are likely to have higher literacy level.

Two of the domains in the PROMS produced a Cronbach’s alpha value of 0.542–0.610. However, when all three domains were combined, the overall Cronbach’s alpha was 0.695. This indicates that the first three domains in the PROMS can be used as a whole to assess the prescribing readiness of medical students.

Test-retest showed that the PROMS has achieved stable reliability as 25/26 items showed no significant difference. In addition, most items either had moderate to good (53.8 %) or fair (42.3 %) correlation. This means that the items in the PROMS were clear enough for the medical students to interpret them in the same way on two different occasions separated by time, thus fulfilling the stability criterion of a good instrument.

Ten out of 26 items were significantly different between the responses of final year medical students from the public and private universities, indicating that the PROMS was able to discriminate between these two groups.

Under the first domain on “undergraduate learning opportunities”, a significantly greater proportion of students from the private university thought that the amount of lectures on basic pharmacology, lectures on the use of drugs in clinical practice (i.e. clinical pharmacology and therapeutics), and small group tutorials about drugs and prescribing was too little as compared to students from the public university. It could be argued that the feedback reflected the nature of the curriculum delivery in each of these medical schools. The medical curriculum of the public university was still more traditional in that, pharmacology was delivered mainly through a block of lecture series interspersed with a few PBL tutorials, as compared to that of the private university where pharmacology lectures were delivered, dispersed within integrated organ system modules. Hence, we were surprised that no significant difference was detected in PBL learning about drugs between students from the public and private universities. This may be due to the inadequacy of pharmacology content delivery during PBL sessions [35, 36] and/or the choices of cases used [8, 35]. These findings have already elicited positive remedies, where additional prescribing workshops, involving inter-professional learning between medical and pharmacy students, are being conducted for medical students in the public university.

Under the domain of “hands-on clinical skills”, a significantly higher proportion of students from the public university (69.9 %) had more experience in preparing and administering a parenteral drug injection as compared to students from the private university (11.1 %). This could be because final year medical students in the public university were required to assist in preparing and administrating parenteral drug injection when there was insufficient staff to carry out these tasks in a certain clerkship. These clinical activities were carried out under the supervision of clinical teachers during that particular clinical clerkship. In contrast, the private university used one of the government hospitals for their clinical training, which have sufficient staff to administer parenteral drugs; and therefore did not utilise medical students for this task.

With regards to the “information gathering behaviours” of medical students, a significantly higher proportion of students from the private university claimed to use all the five listed resources: web based resources, online medical school resources, their own textbook, their own British National Formulary or equivalent, and library resources, on a daily to weekly basis as compared to students from the public university. The basis for this striking difference was not explored in this study, but we speculate that it could be due to the more robust student-centered PBL approach of the private university’s medical curriculum and/or a better information and communication technology support in the private university. In addition, medical students from the public university may tend to depend more on lecture hand-outs than other sources of information. However, this scenario may be fast changing with the availability of WIFI on campus, and the fact that most medical students own a smartphone.

Under the domain on “Factors affecting prescribing skills”, a significantly higher proportion of students from the private university agreed or strongly agreed that there were insufficient lectures or formal teaching on prescribing drugs. This concurred with our findings on the domain of “undergraduate learning opportunities”.

The items in the PROMS instrument assessed the students’ readiness to prescribe in three domains. If students rated that they had sufficient learning opportunities on drugs and prescribing, adequate hands-on clinical skills practice, and the ability to gather information independently, this would infer that they would be more ready to prescribe upon graduation.

In the present study, medical students from both our participating public and private universities felt that their medical programmes did not provide sufficient teaching and learning of prescribing skills. These findings indicate insufficient emphasis on clinical pharmacology and prescribing skills training in the medical curriculum. This may result in poor prescribing habit and may jeopardize patient safety. In response to this concern, many medical schools have introduced educational interventions to improve prescribing competency, such as individual teaching with a supervisor or compulsory workshops prior to their clinical clerkships [37].

### Limitations

Two cohorts were recruited for this study: one cohort to perform the discriminative validity (public university = 123, private university = 53), and the other cohort to perform the test-retest (n = 119, public university). This was because logistically it was not possible to carry out the retest with the first cohort as they were about to complete their exit examination. Another possible limitation is that, although it would be ideal to have an equal number of students recruited from the public and private universities, this in practice is difficult to achieve. This may have affected the ability of the PROMS to compare the responses obtained. However, in both universities, we recruited the entire class of final year medical students.

We were also unable to perform convergent validity, as there were no other validated instruments to assess the prescribing readiness of medical students available at the time of our study.

### Strengths

However, the strength of our study was that we validated the PROMS for face and content validity, construct validity and reliability; as we have not found any other literature that reports the validation of an instrument to assess the prescribing readiness of medical students.

## Conclusions

The PROMS was found to be a reliable and valid instrument for assessing medical students’ readiness to prescribe in Malaysia. It can be used to reflect the adequacy of medical programmes in training students for prescribing. Future studies should look into the prescribing practices of our new graduates to better evaluate the adequacy of the curriculum in training medical undergraduates for the complex task of prescribing.

## Additional file

Additional file 1:
**The Prescribing Readiness of Medical Students (PROMS) Instrument. (PDF 143 kb)**

## Abbreviations

(GMC): General medical council
(NGT): Nominal group technique
(OSCE): Objective structured clinical examination
(PROMS): Prescribing readiness of medical students
(PBL): Problem-based learning
(WHO): World Health Organisation
