# Supplementary material for: Development and validation of an instrument to assess the prescribing readiness of medical students in Malaysia
Source: BMC Med Educ. 2015 Sep 21;15:153. doi: 10.1186/s12909-015-0433-z (PMC4578793; doi:10.1186/s12909-015-0433-z)
Supplement: Additional file 1: — The Prescribing Readiness of Medical Students (PROMS) Instrument. (PDF 143 kb) [file 12909_2015_433_MOESM1_ESM.pdf]

The Prescribing Readiness of Medical Students (PROMS) Instrument

Section A: Your Undergraduate Training Experience on Drugs and Prescribing

(Please tick the appropriate response)

**In relation to the following kinds of undergraduate learning about drugs there was/were**						
**No**	**Statement**	**Far too little**	**Too little**	**About right**	**Too much**	**Far too much**
1	Lectures on the basic pharmacology of drugs					
2	Lectures on the use of drugs in clinical practice					
3	Small group tutorials about drugs and prescribing					
4	Problem-based learning about drugs					
5	Workshops on prescribing issues					
6	Electronic learning opportunities					
7	Others (Specify ___________________________)					

**How many times did you undertake the following clinical skills (with supervision or feedback) during your undergraduate training?**						
**No**	**Statement**	**Never**	**1-5**	**6-10**	**11-15**	**>15**
7	Write a patient's medications in a hospital drug cardex					
8	Calculate drug doses					
9	Set up a drug infusion pump					
10	Prepare and give a parenteral drug injection					
11	Set up and give a bag of intravenous fluid					

**How often did you use the following resources to aid your learning about drugs in your final year?**						
**No**	**Statement**	**Never**	**Yearly**	**Monthly**	**Weekly**	**Daily**
12	Web-based resources (PubMed/BNF/MIMS/Epocrates/Micromedex or equivalent)					
13	Online medical school resources (lecture notes/Computer-aided learning, etc)					
14	Your own textbook					
15	Your own BNF or equivalent (specify:_____________)					
16	Library resources					
17	Others (Specify ___________________________)					

Section B: Factors Affecting Your Learning of Prescribing Skills

(Please tick the appropriate response)

**How much have the following factors affected your undergraduate learning of prescribing skills?**						
**No**	**Statement**	Strongly disagree	Disagree	Somewhat agree	Agree	Strongly agree
1.	No lecture or structured training on the various skills required in prescribing drugs					
2.	The amount of knowledge on drugs needed to be learnt *(e.g. actions, uses, adverse effects and interactions*) is too much during a 6-8 week clinical posting					
3.	We will not be questioned by examiners on prescribing of drugs in the final examination					
4.	Many clinical teachers do not explain the rationale for their choice of drugs prescribed					
5.	Not enough reinforcement of pharmacology knowledge in the clinical years					
6.	It is not necessary for me to know how to prescribe drugs before graduation; I will learn it when I start work					
7.	Lack of consensus among clinical teachers on the choice of drug(s) prescribed					
8.	Preclinical learning of pharmacology didn't have enough clinical relevance, especially with respect to making rational drug choice					
9.	No actual practice (even if under supervision), experience or emphasis on prescribing in clinical years					
10.	Limited access to information on drugs that can help in making rational drug-choice					

What change would you like to see to improve the training of prescribing skills in your institute?

____

____

Any other comments:

____

____

____

THANK YOU AGAIN!
